# Evaluating the potential for prezygotic isolation and hybridization between landlocked and anadromous alewife (*Alosa pseudoharengus*) following secondary contact

**DOI:** 10.1111/eva.12645

**Published:** 2018-06-14

**Authors:** Katherine A. Littrell, David Ellis, Stephen R. Gephard, Andrew D. MacDonald, Eric P. Palkovacs, Katherine Scranton, David M. Post

**Affiliations:** ^1^ Department of Ecology and Evolutionary Biology Yale University New Haven Connecticut; ^2^ Fisheries Division Connecticut Department of Energy and Environmental Protection Old Lyme Connecticut; ^3^ Long Marine Laboratory University of California Santa Cruz Santa Cruz California; ^4^ University of California Los Angeles Los Angeles California

**Keywords:** dam removal, fish passage, habitat connectivity, hybridization, prezygotic isolation, secondary contact

## Abstract

The recent increase in river restoration projects is altering habitat connectivity for many aquatic species, increasing the chance that previously isolated populations will come into secondary contact. Anadromous and landlocked alewife (*Alosa pseudoharengus*) are currently undergoing secondary contact as a result of a fishway installation at Rogers Lake in Old Lyme, Connecticut. To determine the degree of prezygotic isolation and potential for hybridization between alewife life history forms, we constructed spawning time distributions for two anadromous and three landlocked alewife populations using otolith‐derived age estimates. In addition, we analyzed long‐term data from anadromous alewife migratory spawning runs to look for trends in arrival date and spawning time. Our results indicated that anadromous alewife spawned earlier and over a shorter duration than landlocked alewife, but 3%–13% of landlocked alewife spawning overlapped with the anadromous alewife spawning period. The degree of spawning time overlap was primarily driven by annual and population‐level variation in the timing of spawning by landlocked alewife, whereas the timing and duration of spawning for anadromous alewife were found to be relatively invariant among years in our study system. For alewife and many other anadromous fish species, the increase in fish passage river restoration projects in the coming decades will re‐establish habitat connectivity and may bring isolated populations into contact. Hybridization between life history forms may occur when prezygotic isolating mechanisms are minimal, leading to potentially rapid ecological and evolutionary changes in restored habitats.

## 1 INTRODUCTION

In the last three centuries, many anadromous fish populations have declined to <1%–10% of their former abundance (Limburg & Waldman, 2009). Dams have historically been a leading contributor to the loss of anadromous fishes worldwide (Hall, Jordaan, & Frisk, 2012; Liermann, Nilsson, Robertson, & Ng, 2012). For anadromous species, dams can block access to high‐quality spawning grounds, increase competition for available spawning sites, increase migration time, create lethal water conditions during migration, and decrease offspring survival (Gosset, Rives, & Labonne, 2006; Hall et al., 2012; Harnish, Sharma, McMichael, Langshaw, & Pearsons, 2014; Locke, Hanson, Klassen, Richardson, & Aubé, 2003; Zhou, Zhao, Song, Bi, & Zhang, 2014). The multiple negative impacts of dams, combined with other anthropogenic stressors, have contributed to the local extirpation of many anadromous fish species (Beamish & Northcote, 1989; Gao, Lin, Li, Duan, & Liu, 2016; Rolls, Stewart‐Koster, Ellison, Faggotter, & Roberts, 2014). To reverse these impacts, restoration projects are removing dams or installing fishways to restore access to spawning sites. Over 1,300 dam removals and hundreds of fish passage projects have been completed in the United States within the last several decades (Lenhart, 2003; Rivers, 2017; U.S. Fish & Wildlife Service, 2012). Fishway installations and dam removals are completed with the intent of restoring anadromous spawning migrations, but there is little consensus on what constitutes successful restoration and there can be unforeseen impacts on population recovery and local ecosystems (Babbitt, 2002; Bernhardt et al., 2007; Grant, 2001; Hart et al., 2002).

One consequence of restoring habitat connectivity is the increased probability that historically separated populations will come into contact with each other. Such a scenario represents an example of secondary contact, when two evolutionary diverged lineages come into contact after a period of geographic isolation (Hutchings & Myers, 1985; Jones, Brown, Pemberton, & Braithwaite, 2006; Tulp et al., 2013). The ecological and evolutionary processes that occur at the very onset of secondary contact can be complex and determine whether populations undergo speciation, fuse into a single population, or create a hybrid zone (Barton & Hewitt, 1989; Coyne, 1992; Hewitt, 1988). Habitat restoration efforts may provide opportunities to study these processes in natural populations. As restoration efforts seek to reconnect fragmented ecosystems (Baguette, Blanchet, Legrand, Stevens, & Turlure, 2013; Beninde, Veith, & Hochkirch, 2015; Resasco, Bruna, Haddad, Banks‐Leite, & Margules, 2017), instances of secondary contact are likely to become more common. Studying secondary contact as it occurs can provide valuable insight into the biological mechanisms that generate and maintain biodiversity in a wide range of taxa and inform conservation efforts that strive to restore ecosystem connectivity.

Anadromous fishes are an excellent study system for questions pertaining to secondary contact, as their diverse life history forms can become genetically isolated via natural or anthropogenic mechanisms. For example, dam construction can isolate a portion of an anadromous fish population in freshwater, leading to the evolution of an alternate life history that completes its entire lifecycle in freshwater (Berg, 1985; Palkovacs, Dion, Post, & Caccone, 2008; Pearse et al., 2009). The evolution of freshwater resident life history forms is found in numerous taxonomic families, including Salmonidae, Osmeridae, Clupeidae, Gasterosteidae, Petromyzontidae, and Plecoglossidae (McDowall, 1988). Examples of adaptations documented in freshwater resident fish populations include smaller size at maturity, smaller foraging morphology (Jones, Palkovacs, & Post, 2013; Karve, von Hippel, & Bell, 2008; Palkovacs & Post, 2009), alternate reproductive strategies (Campbell, 1977; Closs, Hicks, & Jellyman, 2013; Gulseth & Nilssen, 2001), and a decreased tolerance to salinity (Velotta, McCormick, O'Neill, & Schultz, 2014; Velotta, McCormick, & Schultz, 2015). Taken together, this suite of life history adaptations creates the potential for considerable ecological and evolutionary differentiation between freshwater resident and ancestral anadromous forms. In cases where there are significant morphological, ecological, or genetic differences between populations, secondary contact could result in competition and competitive exclusion (Perry, Feder, Dwyer, & Lodge, 2001), coexistence through niche partitioning and character displacement (Aguilee, de Becdelievre, Lambert, & Claessen, 2011; Levine & HilleRisLambers, 2009; Mayfield & Levine, 2010), speciation via reinforcement (Hasselman et al., 2014), or hybridization (Barton & Hewitt, 1985, 1989; Hewitt, 1988). Secondary contact that results from restoring spatial connectivity could have large impacts on anadromous fish populations, but the outcome depends on the potential for gene flow between anadromous and freshwater resident populations. Gene flow between populations is possible if prezygotic isolating mechanisms such as divergent mating behavior, spatial isolation, temporal isolation, and gamete incompatibility are minimal (Coyne & Orr, 2004).

Species in the genus *Alosa* (shad and river herring) have been identified as the second most prevalent genus in threatened freshwater ecoregions, with 14 of 25 alosine species located in heavily obstructed river systems (Bernhardt et al., 2005; Vörösmarty et al., 2010). In North America, alewife (*Alosa pseudoharengus*) is the target of conservation management plans along the Atlantic coast using fishway installations to restore migratory pathways to historical spawning grounds (Hasselman & Limburg, 2012; Lake, Ravana, & Saunders, 2012). Many of the systems targeted for restoration contain independently evolving populations of landlocked alewife, the freshwater resident form of anadromous alewife, in lakes and reservoirs above the dams (Palkovacs et al., 2008). In lakes in southern Connecticut, landlocked alewife were isolated from anadromous populations 300–500 years ago, likely as a result of colonial dam construction (Palkovacs et al., 2008; Twining & Post, 2013). Landlocked alewife populations have rapidly evolved ecological and evolutionary differences from anadromous alewife during this relatively short period of reproductive isolation (Jones et al., 2013; Palkovacs, Mandeville, & Post, 2014; Palkovacs & Post, 2009; Post, Palkovacs, Schielke, & Dodson, 2008; Schielke, Palkovacs, & Post, 2011).

Here, we ask how variation in spawning time might influence the potential for gene flow between landlocked and anadromous alewife populations. Alewife can hybridize with its sister species, blueback herring (*Alosa aestivalis*) (Hasselman et al., 2014). This suggests a lack of postzygotic isolating mechanisms and a high probability of hybridization between life history forms if there is spawning time overlap. Alewife is a broadcast spawner and does not exhibit complex behaviors during mating that may impede hybridization. In general, anadromous alewife has been reported to spawn earlier (April–June) (Kissil, 1974) than landlocked alewife (May–August) (Nigro & Ney, 1982). Therefore, divergence in spawning time may create prezygotic isolation, which could be an important limiting factor for hybridization. We used otolith‐based age estimates and migration data to quantify the timing of reproduction and explore sources of variation in spawning period that may contribute to or inhibit hybridization between alewife life history forms.

## 2 MATERIALS AND METHODS

### 2.1 Data collection

#### 2.1.1 Migration time

We acquired adult anadromous alewife migration data from three fishways in Connecticut to examine historical trends in anadromous alewife spawning behavior. The Connecticut Department of Energy and Environmental Protection (CT DEEP) collected the data across multiple years. The fishways were located in the Branford Supply Ponds (2006–2016), Mill Brook leading into Rogers Lake (2002–2016), and Bride Brook leading into Bride Lake (2003–2016). Daily fish counts of adult anadromous alewife passing through the fishway into the lake and water temperature were collected by CT DEEP 5–7 days a week and recorded until spent adult fish began returning to sea, at which point the fish counters were removed. Due to the removal of the fish counter and termination of fish counts with outmigration of adults, we excluded the upper and lower 5% of the run, constraining the data to the middle 90% of the run (based on the total number of fish passing through the fishway). We acquired daily ocean surface temperature data (New London, CT) from NOAA's National Centers for Environmental Information water temperature database (NOAA Tides and Currents, 2018).

#### 2.1.2 Spawning time

We estimated alewife spawning time in five lakes in southern Connecticut. All of the systems sampled are within 25 km of the coast (see Palkovacs et al. (2008) for a map of the study sites). Bride Lake (41.3276°N, 72.2378°W) and Dodge Pond (41.3275°N, 72.1986°W) are spawning grounds for anadromous alewife and support young‐of‐the‐year (YOY) alewife for the period of time from hatching in the spring to marine migration in the fall. Pattagansett (41.3728°N, 72.2312°W) and Quonnipaug (41.3889°N, 72.6986°W) Lakes are populated by landlocked alewife, with access from the ocean blocked by dams. Rogers Lake (41.3637°N, 72.3000°W) is populated by landlocked alewife, but a recent fishway installation in 2014 and anadromous alewife stocking program reintroduced anadromous alewife into Rogers Lake. Thus, Rogers Lake is the site of ongoing secondary contact between alewife life history forms. A future secondary contact event is likely to occur in Pattagansett Lake, which is under consideration for a fishway installation within the coming decade. Pattagansett Lake and Dodge Pond are within the Pattagansett River watershed and anadromous alewives genetically similar to those in Dodge Pond may eventually populate Pattagansett Lake.

Fish were captured from all five lakes between July 27 and August 21 (2013, 2014, 2015) using a small research purse seine (4.87 m deep ×35.36 m long, mesh size 1/16 inches) designed to encircle 100 m^2^. We collected 50–150 fish in a single night from the pelagic and littoral zones in six separate seine sets performed in different locations across the lake (Table 1). All fish were immediately euthanized using MS‐222 and stored at −20°C until processing. To quantify the potential for prezygotic isolation between anadromous and landlocked alewife, we used otolith‐derived age estimates to develop spawning time distributions for all five lakes (two anadromous, three landlocked populations). For Rogers Lake, no adult anadromous alewife passed through the fishway into the lake in 2014. In the spring of 2015, Rogers Lake was stocked with 130 adult anadromous alewife from Mill Brook. Based on this very small number of adult anadromous alewife from Mill Brook, we believe it is unlikely that we captured YOY anadromous alewife in 2015. Therefore, we treated Rogers Lake in 2014 and 2015 as purely landlocked populations. To supplement the anadromous alewife population in Rogers Lake, adult anadromous alewife from Bride Lake have been stocked into Rogers Lake each year starting in 2016.

**Table 1:** Alewife sampling information and age estimation using a length–age regression

Lake	Year	Sampling date	Fish total length (mm)	Fish aged by otoliths	Fish aged by regression	Length–age regression equation	*R* ^2^
Bride	2014	5 Aug	33–68	104	0	*y* = 47.81 + 0.579*x*	0.28
Bride	2015	4 Aug	35–66	100	0	*y* = 55.28 + 0.349*x*	0.14
Dodge	2014	4 Aug	31–55	100	0	*y* = 41.89 + 0.834*x*	0.26
Dodge	2015	3 Aug	30–54	99	0	*y* = 45.30 + 0.731*x*	0.25
Pattagansett	2014	7 Aug	25–85	79	9	*y* = 2.69 + 0.904*x*	0.89
Pattagansett	2015	6 Aug	33–82	102	13	*y* = −4.20 + 1.067*x*	0.87
Quonnipaug	2014	21 Aug	18–91	100	65	*y* = 11.19 + 0.712*x*	0.89
Quonnipaug	2015	7 Aug	16–85	100	15	*y* = 5.52 + 0.845*x*	0.93
Rogers	2013	13 Aug	24–63	49	71	*y* = 12.40 + 0.882*x*	0.62
Rogers	2014^a^	6 Aug	11–75	21	51	*y* = 12.40 + 0.882*x*	0.62
Rogers	2015	27 July, 5 Aug	20–79	99	286	*y* = 5.13 + 0.894*x*	0.82

a There were too few otoliths from the Rogers 2014 sampling season to accurately estimate age. We used the length–age regression from 2013.

We estimated alewife hatch date by counting otolith daily growth increments. Both sagittal otoliths were removed and mounted on a glass microscope slide with a heat malleable plastic resin. Only 20 otoliths were available from Rogers Lake in 2014. We used the length‐age regression from the Rogers Lake 2013 population to estimate age in 50 additional Rogers Lake 2014 fish. We polished opaque otoliths using 1,000 and 2,000 grit automotive sanding paper and fine polished the surface using a 0.05 μm slurry of alumina powder. Increments were counted a total of five times by the same observer under 20× to 40× magnification (Leica DM LS2). We excluded samples that were inconsistent across counts, typically with differences >5–7 days between individual counts. Approximately 100 random fish per lake, including the largest and smallest individuals, were counted to capture the full range of the spawning period. In some years, <100 fish were available due to low capture rates in the field and damage to the otoliths during extraction, particularly in fish <20 mm. For landlocked fish, there was a strong relationship between length and age (Table 1). We used the age–length regression equation for each lake and year to estimate the age of additional fish and replace fish with damaged otoliths. Only 20 otoliths were available from Rogers Lake in 2014. We used the length–age regression from the Rogers Lake 2013 population to estimate age in 50 additional Roger Lake 2014 fish. Anadromous fish did not exhibit a strong relationship between length and age; therefore, we did not use length–age regressions to estimate hatch date in additional fish.

Spawning date was calculated by combining data on otolith‐derived hatch dates with temperature‐derived estimates of growth rate during development within the egg. We used temperature profiles from biweekly water sampling at the deepest part of each lake to interpolate daily water temperatures throughout the spawning season. The first 3 m of the epilimnion was averaged to approximate the temperature in benthic habitat where alewife spawn. Our formula for calculating development time in the egg from lake temperatures, *T = *114.05e^−0.048F^ (*R* ^2^ = 0.94), was derived by digitization of the data presented in figure 3 of Edsall (1970), where *T* is days to hatching and *F* is temperate in degrees Fahrenheit. The egg development time was added to our otolith‐derived hatch times to determine days since spawning. The number of days since spawning was subtracted from the date of capture to calculate a spawning date for each fish. All fish were collected under CT DEEP Scientific Collector's Permits SC‐11016 and SC‐14023 and handled in compliance with Yale's Institutional Animal Care and Use Committee protocols 10734 (2012) and 10734 (2015).

### 2.2 Statistical analysis

#### 2.2.1 Migration data

As the anadromous alewife spawning migration (movement upriver) occurs in intermittent pulses over the spring, we isolated several metrics as indicators of the overall timing of migration: the day on which 10%, 50%, or 90% of the population had migrated and the day of peak (maximum) migration. We used a linear regression with Gaussian distributed errors (Bates, Mächler, Bolker, & Walker, 2015; R Core Team, 2017) to estimate the relationship between the timing of migration and ocean water temperature, while accounting for any trend across years and individual population effects. We treated all factors as fixed effects, including the differences between populations because of the low replication of rivers (*n* = 3). We also included a second‐order ocean temperature term that created a quadratic relationship with a single peak for migration. This term allowed for the possibility of a thermal optimum for migration, where migration would decrease at warmer or colder temperatures. We performed model selection using AIC to determine the order of the relationship of migration with ocean temperature and whether any covariates explained the variation in the relationship between migration and ocean temperature. We also used a simple linear regression (Bates et al., 2015; R Core Team, 2017) to determine whether the size of the migratory run (the total number of fish observed) predicted the overall duration of the migration period.

#### 2.2.2 Spawning data

Anadromous and landlocked alewife spawning occurs from April to mid‐July, creating a set of unimodal distributions of spawning dates that differ by alewife life history form, lake, and year. We modeled the distribution of spawning dates using time‐to‐event analysis, commonly used in survival analysis. Time‐to‐event models assume some inherent variability in the timing of the event; individuals experience the event at times distributed according to a probability distribution (Hougaard, 2000). The instantaneous probability of the event (the hazard function) can be affected by covariates making individuals with certain traits or in certain groups more or less likely to experience the event earlier or later.

We generated event (spawning) times for each individual, defined as the time from January 1 to the estimated spawning date. As the resolution of our estimates of spawning time was on the order of a day, we used interval censoring to account for the fact that spawning may have occurred at any time during the day (but taking the exact event time as the midpoint of the day did not affect our results). We assumed spawning times followed a Weibull distribution with shape and scale parameters that allow flexibility in the shape of the underlying instantaneous probability of spawning (hazard function). We hypothesized that spawning may be affected by alewife life history form, while accounting for differences between years and between lakes.

We considered lake temperature as a factor, but water temperature in our five lakes increased linearly over the summer, making it impossible to separate the effects of time from the effects of water temperature (Figure A1 in Appendix S1). Some alewife populations spawned earlier in cooler water, and some spawned later in warmer water. Water temperatures in the five lakes were very similar, and landlocked lakes were not more similar to each other than they were to anadromous lakes. Thus, we excluded lake temperature from our analysis, supported by the fact that lakes varied only very slightly, and not systematically, in temperature (see Appendix S1 for more detail).

We approximated and maximized the likelihoods in R with original code (R Core Team, 2017) and performed model selection with AIC (Appendix S1). This let us estimate the parameters of the distribution of spawning times (the shape and rate parameters) as well as the coefficients of the covariates: alewife life history form, lake, and year. From these estimates, we calculated the mean and variance of the estimated spawning time distributions, as well as the probability of both life history forms spawning at the same time (Appendix S1). We estimated the potential for hybridization using two metrics: the percent of the population expected to experience an “interbreeding event” and the “spawning overlap.” The percent of the population expected to experience an interbreeding event was derived from the cumulative probability that one anadromous and one landlocked alewife spawn on the same day throughout the entire spawning season. Spawning overlap was defined as the percentage of the landlocked population that spawns at any point in time within the anadromous spawning distribution, but not necessarily on the same day. Neither measure of hybridization potential makes any assumptions about sex ratios or spawning behavior. Anadromous populations were treated as a single “anadromous” spawning time distribution due to their nearly identical distributions and for comparisons with landlocked populations. The Rogers Lake and Quonnipaug Lake landlocked populations were also treated as one spawning time distribution because they did not differ statistically from one another. We compared the Pattagansett Lake landlocked spawning time distributions to the anadromous distribution independently because they were statistically different from the other two landlocked alewife populations in Rogers Lake and Quonnipaug Lake.

## 3 RESULTS

### 3.1 Migration

**Figure 1:**
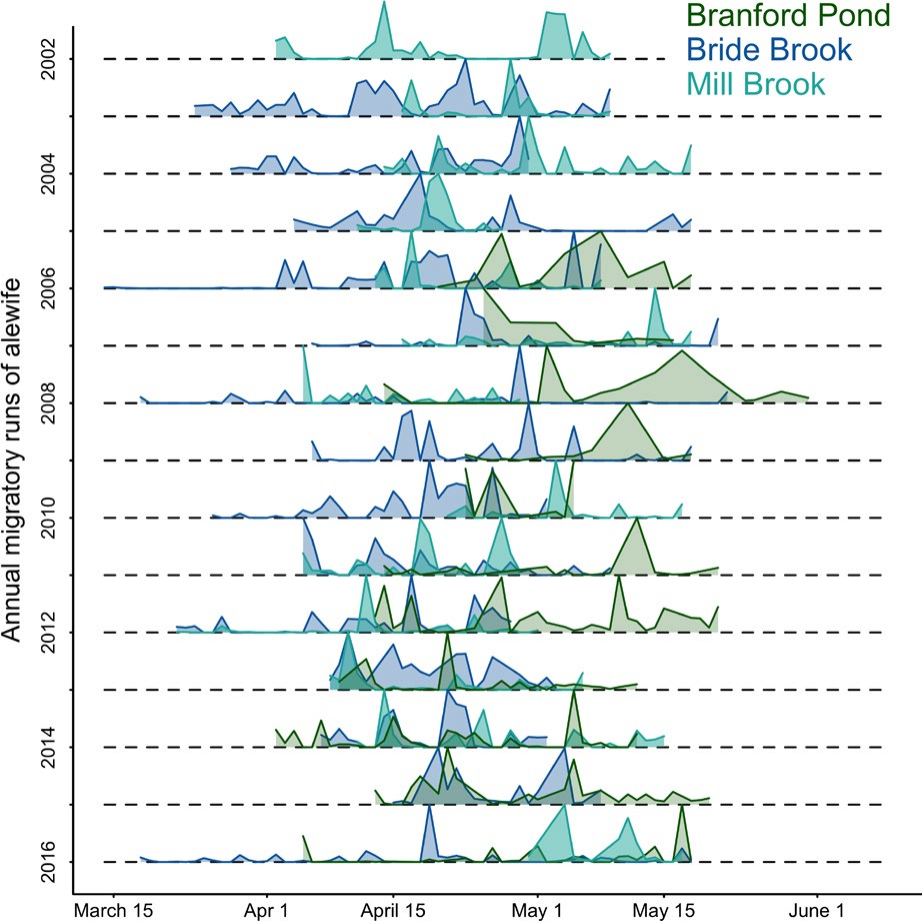
Probability distributions of adult anadromous alewife migration by arrival date from 2002 to 2016 in Bride Brook, Mill Brook, and Branford Supply Ponds. The probability distributions are based on CT DEEP counts at each location using electronic fish counters. The annual number of anadromous alewife passing through each fishway is provided in Table B1 in Appendix S2

Adult anadromous alewife generally migrated from mid‐March to mid‐May, with anadromous alewife starting to arrive on the spawning grounds in small numbers as early as late February (Figure 1). The majority of the anadromous alewife migration occurred within a 20‐ to 30‐day period beginning the second week of April. Across the four migratory timing metrics, the models that were highly supported included ocean surface temperature and migratory river, indicating that there was a significant relationship of migration to ocean temperature, but that it varied across populations. Models with a second‐order term for ocean temperature were equally as likely as those with a linear relationship, indicating no support for a thermal optimum for migration (Appendix S3). We found no evidence for a secular change in the timing of migration; year was not a factor in the most highly supported models. Although not significant, the anadromous alewife run at Bride Brook tended to arrive 15–20 days earlier than anadromous alewife in Mill Brook and Branford Supply Ponds. Anadromous alewife migrated when ocean surface temperatures were between 4.9 and 16.0°C, with average migration temperatures between 7.0 and 12.0°C.

Run size (number of spawning adults) at Bride Brook over the last decade ranged from 68,731 to 354,862 fish, with a mean of 162,075 ± 94,588 fish. The Mill Brook had runs ranging from 99 to 15,362 fish (mean = 5,620 ± 5,333 fish), and the run at the Branford Supply Ponds ranged from 563 to 50,668 fish (mean = 8,297 ± 17,179 fish). Run size negatively correlated with run duration at Bride Brook (*F*_1,11_ = 9.518, *p* = 0.01, *R* ^2^ = 0.4151), but there was no relationship between run size and run duration at Mill Brook or the Branford Supply Ponds, likely due to smaller run sizes.

### 3.2 Spawning

**Figure 2:**
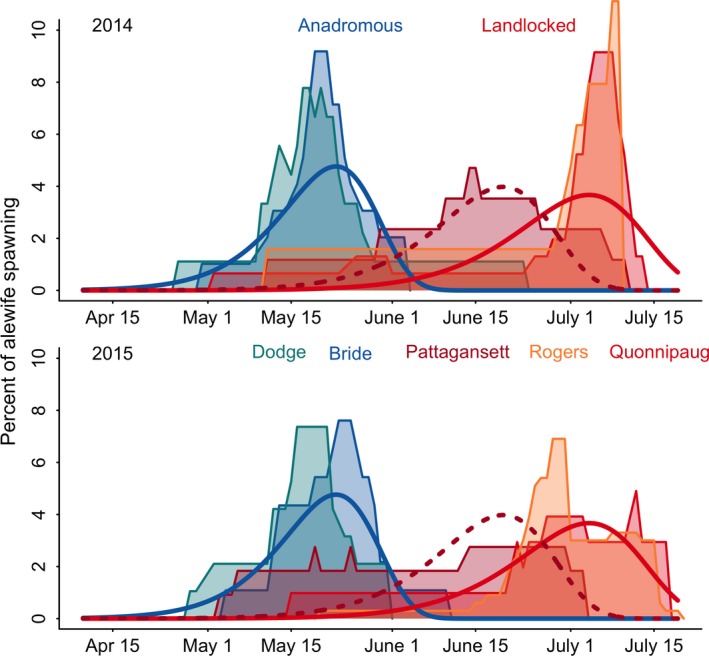
Probability distributions of spawning time for Bride/Dodge anadromous (blue solid line), Rogers/Quonnipaug landlocked (red solid line), and Pattagansett landlocked (red dotted line) alewife from 2014 to 2015. The colored probability distributions represent otolith‐derived spawning dates for each population

Landlocked alewife spawned later and over a longer duration than anadromous alewife. Landlocked alewife also had greater variation in spawning date than anadromous alewife (Figure 2). Life history form was the most important explanatory variable for spawning time in alewife, but spawning time did differ by year and population (Table 2). The Rogers Lake landlocked population spawned much earlier in 2013 than in 2014 and 2015. The Pattagansett Lake population spawned earlier than all other landlocked populations in both 2014 and 2015.

**Table 2:** Models and AIC selection scores for alewife spawning time

Model	Delta AIC
y ~ form^a^ + 2013 + all lakes	0
y ~ form + all years + Pattagansett Lake	0.466
y ~ form + 2013 + Pattagansett Lake	2.24
y ~ form + all years + all lakes	20.5
y ~ form + Pattagansett Lake	173
y ~ form + 2013	261
y ~ form + all years	263
y ~ form	391
y ~ form + all lakes	749
y ~ all years + all lakes	765
y ~ 1	2,057

a Form = alewife life history form.

Anadromous alewife spawned over a period of 35–40 days, beginning as early as April 26 and continuing until the first week of June. The water temperature during this time period ranged from 12.5 to 21.2°C. Mean spawning date for anadromous alewife was May 19 ± 9.3 days (Table 3). Peak spawning occurred in mid‐May when water temperatures reached 17.0–20.0°C. There were subtle differences in the spawning periods of anadromous populations, with Dodge Pond alewife initiating and reaching peak spawning up to a week earlier than alewife in Bride Lake. Although spawning duration was nearly identical in both populations, in Dodge Pond 2014, we detected one fish that hatched in late June. This individual extended the estimated spawning period from 38 to 59 days; this was the only fish in the sample that hatched after 2nd June. We detected a 2‐week delay between the end of migration in Bride Lake and peak spawning activity, which was similar to other anadromous alewife populations (Rosset et al., 2017).

**Table 3:** Mean spawning date for anadromous (A) and landlocked (L) alewife populations

Population	Mean spawning date	SD (days)
2014/2015 Bride and Dodge (A)	May 19	9.3
2014/2015 Rogers and Quonnipaug (L)	June 29	12.1
2014/2015 Pattagansett (L)	June 15	11.1
2013 Rogers (L)	June 15	11.1

Landlocked alewife populations started spawning in early to late May and continued spawning for approximately 60 days, when water temperatures were between 13.5 and 27.5°C. The Pattagansett Lake and Quonnipaug Lake populations commenced spawning between May 2 and May 15, while the Rogers Lake population started spawning slightly later from May 11 to May 20 depending on the year. The Pattagansett Lake population reached peak spawning in mid‐June (22.0–25.0°C), 2–3 weeks earlier than all other landlocked lakes. Peak spawning in Quonnipaug Lake occurred in early to mid‐July (25.0–27.0°C). In the Rogers Lake 2014 and 2015 populations, peak spawning also occurred from early to mid‐July (25.0–27.0°C), but peak spawning occurred in mid‐June during the 2013 spawning season (22.0–24.0°C).

We detected low, but variable levels of spawning time overlap and interbreeding events between anadromous and landlocked alewife. Our model‐derived spawning overlap estimate, considering all populations and years, was <15%. The degree of overlap varied by year and population, with some landlocked populations in certain years overlapping more with anadromous alewife than others. Approximately 3% of landlocked alewives in Rogers Lake and Quonnipaug Lake were predicted to have spawned within the anadromous alewife spawning period. Spawning overlap was higher in Pattagansett Lake at 13%. Our data typically followed the model with the exception of the Pattagansett Lake 2015 population. In 2015, the data for Pattagansett Lake deviated from our spawning model and indicated that spawning overlap with anadromous alewife was as high as 30%. The spawning overlap estimates were relatively liberal, as they did not consider what day both alewife life history forms spawned. A more conservative estimate of the hybridization potential was the percentage of the landlocked population that experienced interbreeding events, or the proportion of the population that spawned on the same day as anadromous alewife in a single spawning season. Our model indicated that 0.1% of the Rogers/Quonnipaug Lake landlocked population and 0.4% of the Pattagansett Lake landlocked population spawned on the same day as anadromous alewife. In both landlocked populations, interbreeding events with anadromous alewife were most likely to occur during the last week of May.

## 4 DISCUSSION

Re‐establishing ecosystem connectivity, which may bring historically isolated populations into secondary contact, has become a common objective in conservation and restoration planning. For anadromous fish, increasing habitat connectivity as a result of dam removals and fishway installations will bring anadromous populations into secondary contact with previously isolated and independently evolving landlocked populations. Variation in reproductive timing between anadromous and landlocked populations will influence the potential for hybridization and level of introgression between life history forms during contact. For alewife in our study lakes, the probability of concurrent spawning between life history forms was low, but variable, due to strong temporal differences in spawning behavior. Landlocked alewife reached peak spawning 1.5 months later than anadromous alewife, but the landlocked alewife spawning distributions did overlap with the anadromous alewife spawning distribution, with 3%–13% of landlocked alewife spawning during the anadromous alewife spawning period. For the Rogers Lake landlocked alewife population, which is experiencing secondary contact with anadromous alewife from Bride Lake, 0.1% of landlocked alewife spawned on the same day as anadromous alewife. Pattagansett Lake landlocked alewife will experience secondary contact with anadromous alewife genetically similar to alewife in Dodge Pond after a fishway installation in the next decade. Due to earlier spawning, 0.4% of Pattagansett Lake landlocked alewife spawned on the same day as anadromous alewife from Dodge Pond. Our model indicated that 13% of the Pattagansett Lake landlocked alewife population spawned within the anadromous alewife spawning period, but the data suggested that this estimate may be as high as 30%. Spawning time overlap and the potential for hybridization between life history forms are largely dependent on the spawning behavior of the particular landlocked populations under consideration. We anticipate that anadromous populations may be more similar in their spawning behavior due to a shared marine environment and similar migratory constraints, while landlocked spawning patterns are subject to local selection pressures and free to diverge from one another across lakes. Our results support this idea.

Our anadromous alewife spawning dates and temperatures are comparable to that of other anadromous alewife populations along the Atlantic coast (Rosset et al., 2017). The anadromous alewife population at Bride Lake has been studied repeatedly, with a focus on the timing of adult anadromous alewife migration. Kissil (1974) observed anadromous alewife migrating from March to June when stream temperatures reached 4.0–5.0°C and adults remained in the lake to spawn anywhere from 3 days to 3 months after arrival. Ellis and Vokoun (2009) identified a stream temperature of 9.0°C as indicative of the start of migration and 13.0°C as the strongest predictor of average run time in three Connecticut anadromous alewife populations, including Bride Lake. Our data indicated that anadromous alewife migrated from March to June when stream temperatures were between 6.0 and 22.0°C. Peak migration occurred from mid‐April to mid‐May when stream temperatures averaged between 11.0 and 14.0°C. Anadromous alewife spawned in Bride Lake and Dodge Pond in the last week of April and peak spawning occurred in mid‐May when lake temperatures reached 17.0–20.0°C. We did not detect the effect of climate change on advancing the arrival date of adult anadromous alewife in Connecticut predicted by Ellis and Vokoun (2009), nor did we identify the Bride Brook population as arriving significantly earlier than other anadromous alewife populations. We note that our results differ because we restricted our dataset to the last 15 years and did not use the weighted‐mean migration temperature in our analysis. We also used the first 5% of the cumulative run total to define run initiation.

Landlocked alewife populations along the Atlantic coast and Laurentian Great Lakes region spawn primarily in late May through July (Gross, 1959; Lackey, 1970; Odell, 1934; Rothschild, 1966), but the spawning season is variable and can extend into August (Pritchard, 1929). Landlocked alewife in Claytor Lake, Virginia, spawned from early May once water temperatures reached 16.0–18.0°C, until early August when temperatures reached 24.0–27.0°C. Peak spawning occurred in the third week of May when temperatures were between 21.0 and 23.0°C (Nigro & Ney, 1982). Nigro and Ney (1982) noted that during this 1974 spawning season, the Claytor Lake landlocked alewife population spawned a month earlier and 4–9 weeks longer than northern landlocked alewife populations. We observed similar spawning patterns to other northern lakes in Rogers, Quonnipaug, and Pattagansett Lakes. For most lakes and years, landlocked alewife spawning started in mid‐May and continued through July at temperatures between 13.5 and 27.5°C. Peak landlocked alewife spawning occurred when temperatures reached 22.0–27.0°C.

The difference in spawning time between anadromous and landlocked alewife may be driven by selective constraints related to migration. Anadromous alewives mature along the Atlantic coast several hundred kilometers or more from their spawning grounds (Neves, 1981). Alewife, as with other anadromous species, relies on internal and external cues at sea to correctly time migration to maximize its survival en route to spawning sites (Berdahl, Westley, Levin, Couzin, & Quinn, 2016; Hansen, Jonsson, & Jonsson, 1993; Quinn & Adams, 1996). Anadromous fishes face the added challenge of timing migration to align with peak conditions for offspring survival. Ideal conditions for migration and spawning may not coincide; a trade‐off may exist between ideal timing of migration and spawning that determines the actual timing of reproduction (Quinn, McGinnity, & Reed, 2016). Environmental conditions such as water temperature and stream discharge have been correlated with migratory timing in anadromous species, particularly salmonids (Hodgson & Quinn, 2002; Quinn & Adams, 1996; Quinn, Hodgson, & Peven, 1997). Spawning sites can become inaccessible or travel conditions lethal during periods of low stream discharge and high temperatures (Goniea et al., 2006; Rand et al., 2006). Iteroparous anadromous species suffer the added constraint of successfully migrating back to sea after spawning. In contrast, landlocked fish are present in the spawning habitat year round, releasing them from many of the selective constraints associated with migration. Landlocked alewife may closely track environmental cues and time spawning to coincide with peak conditions for offspring survival (Lyons et al., 2015). This time period may be much later, and at warmer temperatures, than the period safe for the migration of anadromous alewife.

A hypothesis for the observed differences in spawning time variability between anadromous and landlocked alewife is there are differences in temperature variability between marine and freshwater ecosystems. Oceans exhibit less temperature variability through time than small bodies of freshwater on continents (Cyr & Cyr, 2003). Landlocked alewives have to be adaptable to rapidly fluctuating environmental conditions in comparison with anadromous alewives in a more constant marine growth environment, which ultimately influences sexual maturation, migration, and spawning (Friedland, 1998; Gardner, 1976).

Our data indicated that changes in spawning time overlap between alewife life history forms were caused by yearly and population‐level variation in landlocked spawning behavior. The sources of this variation are unknown, as we did not detect any significant environmental differences, most notably lake temperature, between our three landlocked lakes. Instead, genetic differences between populations, resulting from natural selection or genetic drift, could drive spawning time variability. All three of our landlocked lakes are genetically isolated and have evolved independently from each other (Palkovacs et al., 2008). Our landlocked alewife populations are as genetically divergent from each other as they are from their ancestral anadromous alewife populations (Palkovacs et al., 2008). Unlike anadromous alewife populations, which exchange genes over a broad marine region (Palkovacs et al., 2014) and are only subject to selection in freshwater lakes for a few months a year, landlocked alewife populations are genetically isolated in freshwater lakes year round and free to adapt to local conditions (Jones et al., 2013; Palkovacs et al., 2014; Palkovacs & Post, 2009; Post et al., 2008; Schielke et al., 2011).

Our data on spawning date may be affected by in‐lake YOY mortality and the outmigration of anadromous alewife. Although we included both the largest (oldest) and smallest (youngest) alewife in each sample, we may have missed a portion of the anadromous YOY cohort that migrated from the lake starting in early June; therefore, it is possible that the anadromous alewife spawning period starts earlier than indicated. We also did not correct for in‐lake mortality in either population and limited our sampling to a single date for each lake per year. As a result, the peak spawning time for both life history forms may be earlier than estimated. We believe that the region of spawning time overlap between life history forms, comprised of the late portion of the anadromous alewife spawning distribution and early portion of the landlocked alewife spawning distribution, is unlikely to be strongly affected by any sampling bias. Our estimated probabilities of spawning overlap may be underestimates, but unlikely to be overestimates, of true spawning potential; what we provide is a conservative estimate of hybridization potential.

The degree of prezygotic isolation between landlocked and anadromous alewife will influence the genetic and ecological outcomes of secondary contact after fish passage river restoration projects. We detected low levels of spawning time overlap between anadromous alewife and two of our landlocked alewife populations (Rogers Lake and Quonnipaug Lake). Less than 3% of landlocked fish in these two lakes spawned at the same time as anadromous alewife. However, among‐lake and among‐year variation in landlocked alewife spawning patterns can increase spawning time overlap to 13%. Our data from Pattagansett Lake in 2015, which deviated from our model, suggested that this upper estimate may be as high as 30%. A spawning time overlap of 3%–13% provides some potential for hybridization and introgression between life history forms. Our spawning time models do not include any behavioral changes that may increase the hybridization potential between anadromous and landlocked alewife populations. It is possible that the presence of large fertile anadromous females and anadromous spawning activity may trigger landlocked alewife to reproduce earlier than expected and increase spawning time overlap higher than predicted in this analysis (Hobbs, Munday, & Jones, 2004).

We cannot predict how hybridization will impact alewife populations long term. Possible outcomes of hybridization include the introduction of maladaptive genes (Glover et al., 2017), production of competitively superior hybrids (Perry et al., 2001), formation of polymorphic populations (Riva‐Rossi, Pascual, Babaluk, García‐Asorey, & Halden, 2007), and speciation via character displacement (Hasselman et al., 2014). It is possible that the introduction of maladaptive traits from landlocked populations (Velotta, McCormick, Jones, & Schultz, 2018; Velotta et al., 2014, 2015) into anadromous populations could reduce anadromous alewife migration success and fitness in marine ecosystems. Changes in alewife morphology and behavior, however, can have immediate effects on freshwater ecosystems. Both alewife life history forms are known for their strong and divergent roles in shaping zooplankton community structure in freshwater lakes. The divergent zooplankton communities are the result of differences in alewife life history (specifically freshwater residency time of YOY), foraging behavior, and morphology (Jones et al., 2013; Palkovacs & Post, 2009; Post et al., 2008). Hybridization between anadromous and landlocked alewife may result in offspring with intermediate size, foraging morphology, and mixed migratory strategies. Due to the strong ecological effects of alewife on freshwater zooplankton community structure, differences in traits among hybrid alewife have the potential to cause direct and rapid changes in the ecology of freshwater lakes.

There are many anadromous fish species facing changes in habitat connectivity as fish passage projects accelerate in the coming decades (Pohl, 2002). Many of these species also have isolated landlocked populations behind migratory barriers (Apgar, Pearse, & Palkovacs, 2017; Berg, 1985; Palkovacs et al., 2008; Pearse et al., 2009), and it is unknown how secondary contact between life history forms will affect populations of conservation concern. When prezygotic isolation prevents gene flow, it is possible for speciation to occur. In contrast, gene flow may result in complex outcomes ranging from the fusion of populations to the formation of a stable hybrid zone (Barton & Hewitt, 1989; Coyne, 1992; Hewitt, 1988). Ecological interactions, such as competition, combined with introgression between life history forms, may have significant implications for the evolution, ecology, and management of anadromous fish populations during secondary contact with freshwater resident populations.

Little is known about the success of all types of river restoration projects or how they affect ecosystems due to a lack of long‐term monitoring (Babbitt, 2002; Bernhardt et al., 2007; Grant, 2001; Hart et al., 2002). Pressing questions include identifying the historical impacts of dams on ecosystems and predicting the impact of dam removals or fish passage projects on future ecosystem function. Part of the ecological response will involve evolutionary processes, including secondary contact. In this manner, fish passage river restoration projects are an ecosystem‐level experiment to test how changing habitat connectivity impacts ecological and evolutionary dynamics.

Restoration projects that aim to re‐establish landscape connectivity may commonly drive secondary contact between historically isolated populations in a variety of taxa. Similar to fishway installations and dam removals in aquatic ecosystems, the construction of wildlife corridors to connect isolated populations in terrestrial ecosystems also alters habitat connectivity (Beier & Noss, 1998). Adaptation to different ecological conditions in isolated habitats is likely to result in the evolution of divergent traits (Bicudo, Anciães, Benchimol, Peres, & Simões, 2016; Fraser, Debes, Bernatchez, & Hutchings, 2014; Santos & Araújo, 2015; Zastavniouk, Weir, & Fraser, 2017). Trait differences between populations can lead to ecological and evolutionary interactions after secondary contact that have the potential to influence the outcome of restoration and conservation projects. Changes in habitat connectivity are not limited to restoration projects. Anthropogenic changes to species ranges (Potts et al., 2014), the spread of invasive species (Kovach et al., 2015; Perry et al., 2001; Zaccara, Antognazza, Buonerba, Britton, & Crosa, 2014), and pollution (Seehausen, van Alphen, & Witte, 1997) are breaking down reproductive barriers and bringing previously isolated populations or closely related species into secondary contact. Using restoration projects as a model to study the ecological and evolutionary dynamics of secondary contact will help inform future conservation and restoration efforts as anthropogenic changes to habitat connectivity accelerate in the coming decades.

## CONFLICT OF INTEREST

None declared.

## DATA ACCESSIBILITY

Data for this study are available from the Dryad Digital Repository: https://doi.org/10.5061/dryad.7qt4220.
