# Supplementary material for: Evaluating the potential for prezygotic isolation and hybridization between landlocked and anadromous alewife (Alosa pseudoharengus) following secondary contact
Source: Evol Appl. 2018 Jun 14;11(9):1554–66. doi: 10.1111/eva.12645 (PMC6183454; doi:10.1111/eva.12645)
Supplement: Supplementary file 1 [file EVA-11-1554-s001.docx]

**Appendix S1: Spawnng Time Model**

Alewife spawning occurs throughout the summer, creating unimodal distributions of spawning dates that differ by alewife life history form, lake, and year. We modeled the distribution of spawning dates using time-to-event analysis, commonly used in survival analysis, where all individuals’ times until the event follow a probability distribution f(x) (Hougaard, 2000). Taking spawning as the event, we generated event times for each individual, defined as the time from January 1 to the estimated spawning date. We assumed spawning times followed a Weibull distribution:


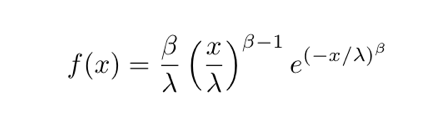


with shape β and scale λ parameters that allow flexibility in the underlying instantaneous probability of spawning (hazard function).

In time-to-event analysis, covariates affect the hazard rate, or instantaneous rate of occurrence: the instantaneous probability of spawning (Sun, 2006).

*λ = exp(ln(λ_0_ +* ***CX****)*

where ***CX*** *= c_1_x_1_ + … + c_n_x_n_* for any covariates. These covariates, or fixed effects, describe the differences between individuals in different groups. We hypothesized that the spawning hazard may be affected by alewife life history form, while accounting for differences between years and between lakes. We also considered lake water temperature as a predictor of spawning behavior. However, lake temperature increased linearly over the spawning season. The day of the year alone explained 89% of the variation in temperature (R^2^=0.8869, slope = 0.15, p < 0.001, Fig. A1).

We also considered that differences in water temperature between lakes or between years might be affecting differences in spawning time. There were significant but very small differences in slope between some years and some lakes, mostly driven by a warm 2015 spring. We excluded lake temperature from our analysis of spawning time, supported by the fact that lakes varied only very slightly, and not systematically, in temperature.

Figure A1: Seasonal lake water temperature for 5 lakes (colored red to blue as in main text) in 2013 (dashed line), 2014 (dotted lines), and 2015 (solid lines).

We treated year as a continuous variable and a fixed effect to determine if there was any trend in spawning date through time. Conceptually, the lake could have been a random effect because we believed that these lakes were a random sample from the larger collection of lakes in the area. However, we had only five lakes in total, and only two anadromous lakes. The small sample size made it prudent to treat lake as a fixed effect, allowing that we may have had an uneven sample across all of the possible random differences between one lake and the overall average of lakes.

Since the resolution of our estimates of spawning time was on the order of a day, we used interval censoring to account for the fact that spawning may have occurred at any time during the day.

We approximated and maximized the Likelihoods in R with original code and performed model selection with AIC.

We calculated two metrics to quantify the hybridization potential between life history forms: the proportion of the population experiencing an “interbreeding event” and the “spawning overlap”. Interbreeding events were defined as the probability that one anadromous and one landlocked alewife spawn at the same time. We took the sum of all interbreeding events across the entire spawning season to determine the proportion of the landlocked alewife population experiencing interbreeding events. The spawning overlap was defined as the as percentage of the landlocked alewife population that spawned within the anadromous alewife spawning period, but not necessarily on the same day.

**Appendix S2: Anadromous Alewife Migration Data**

**Table B1:** Annual number of adult anadromous alewife migrating into Bride Brook, Mill Brook, and Branford Supply Ponds.

| Year | Bride Brook | Mill Brook | Branford Ponds |
| --- | --- | --- | --- |
| 2002 | -- | 14287 | -- |
| 2003 | 117150 | 4198 | -- |
| 2004 | 81350 | 2227 | -- |
| 2005 | 68757 | 1793 | -- |
| 2006 | 129114 | 9093 | 3123 |
| 2007 | 77395 | 99 | 1318 |
| 2008 | 76108 | 698 | 2684 |
| 2009 | 74839 | -- | 3505 |
| 2010 | 164149 | 1213 | 50668 |
| 2011 | 196996 | 8356 | 4553 |
| 2012 | 287003 | 15361 | 613 |
| 2013 | 354862 | 8264 | 563 |
| 2014 | 260926 | 1893 | 1527 |
| 2015 | 218076 | 134 | 538 |
| 2016 | 147552 | 407 | 1512 |

**Appendix S3: Anadromous Alewife Migration Models and Delta AIC Values**

**Table C1:** Delta AIC values: the difference between the AIC of each model and the lowest AIC of those in the suite of models considered. We consider 12 models for each of four metrics.

| Model | Day of 10% | Day of 50% | Day of 90% | Day of peak |
| --- | --- | --- | --- | --- |
| y ~ 1 | 14.65 | 18.47 | 10.74 | 21.74 |
| y ~ oceanT^*^ | 3.62 | 7.67 | 0.82 | 2.52 |
| y ~ oceanT^2^ | 4.88 | 9.49 | 2.45 | 4.32 |
| y ~ year | 16.65 | 20.03 | 12.31 | 22.23 |
| y ~ river | 6.20 | 10.18 | 9.40 | 15.74 |
| y ~ year + river | 8.14 | 10.91 | 10.38 | 14.06 |
| y ~ oceanT + year | 2.92 | 8.39 | 1.36 | 3.05 |
| y ~ oceanT + river | 0.00 | 0.58 | 0.00 | 0.00 |
| y ~ oceanT + year + river | 0.69 | 2.31 | 1.40 | 1.86 |
| y ~ oceanT + oceanT^2^ + year | 4.27 | 10.36 | 3.22 | 4.45 |
| y ~ oceanT + oceanT^2^ + river | 1.30 | 0.00 | 0.72 | 1.96 |
| y ~ oceanT + oceanT^2^ + year + river | 2.02 | 2.00 | 2.48 | 3.76 |

*oceanT = ocean temperature
